# Circadian stage-dependent and stimulation duration effects of transcutaneous auricular vagus nerve stimulation on heart rate variability

**DOI:** 10.1371/journal.pone.0277090

**Published:** 2022-11-03

**Authors:** Duyan Geng, Kai Yang, Zhigang Fu, Yi Zhang, Chao Wang, Hongxia An

**Affiliations:** 1 State Key Laboratory of Reliability and Intelligence of Electrical Equipment, Hebei University of Technology, Tianjin, China; 2 Key Laboratory of Electromagnetic Field and Electrical Apparatus Reliability of Hebei Province, Hebei University of Technology, Tianjin, China; 3 Physical Examination Center of the 983rd Hospital of the Chinese People’s Liberation Army Joint Logistic Support Force, Tianjin, China; University of Pennsylvania Perelman School of Medicine, UNITED STATES

## Abstract

Transcutaneous auricular vagus nerve stimulation (taVNS) can improve autonomic nerve function and is currently undergoing extensive clinical research; however, its efficacy heterogeneity has caused great controversy. Heart rate variability (HRV), a biomarker reflecting autonomic function, exhibits a time-varying pattern with circadian rhythms, which may be the main reason for the inconsistent stimulation effects. To test this conjecture, we performed isochronous acute stimulation experiments at intervals of 12 h. The results showed that HRV indicators representing vagal nerve activity significantly increased when stimulation was performed in the morning, and the enhancement of high frequency continued into the recovery period. However, the evening stimulation did not yield similar results. In addition, we found that improvements in the measures of autonomic balance were more pronounced in the presence of lower vagal activity. By increasing the stimulation duration, we also found that the effect of taVNS on HRV was not regulated by duration; in other words, HRV changes only had the best effect at the beginning of stimulation. These studies allowed us to determine the optimal stimulation phase and duration and potentially screen the optimal candidates for taVNS.

## Introduction

The vagus nerve is the main component of the parasympathetic nervous system and can antagonize the sympathetic nervous system. It not only regulates the body’s breathing, heart rate, and gastrointestinal secretion, but also maintains the balance of the autonomic nervous system. With age and the appearance of certain diseases (e.g., depression, hypertension, or heart failure) [1–3], the autonomic nervous system in humans undergoes various degrees of damage, characterized by increased sympathetic activity and decreased parasympathetic activity [4]. To increase the activity of the vagus nerve and improve the balance of the autonomic nervous system, a new method different from drug therapy has been proposed [5].

Vagus nerve stimulation (VNS) is an FDA-approved treatment for epilepsy and treatment-resistant depression, and following a recently completed study [6], VNS in combination with rehabilitation therapy is also FDA-approved treatment for stroke. However, traditional VNS surgery is invasive, and wearing of the VNS device may result in adverse effects such as pain, coughing, and breathing difficulty, which may limit its use in wider populations [7]. To overcome the limitations of traditional VNS, non-invasive method of vagal nerve stimulation via the auricular branch of the vagus nerve (ABVN) has been developed.

The external ear is the only location on the body where the VN sends its cutaneous branch [8]. Anatomical studies of the ear have shown that the tragus, concha, and cochlea are the sites where cutaneous afferent vagus nerve distribution exists in humans, and stimulation of the auricular branch of the vagus nerve has similar effects to invasive techniques [9, 10]. An fMRI study of human tragus taVNS revealed a similar activation pattern to that of conventional cervical VNS, further supporting the potential of tragus stimulation as a non-invasive alternative [11]. taVNS is a simple, non-invasive, and inexpensive treatment that has shown benefits in extensive clinical studies of stroke, epilepsy, diabetes, and severe traumatic brain injury [12–14]. Given the widespread association of autonomic nervous system damage with numerous diseases, it is critical to investigate the effect of taVNS on autonomic nervous system function.

Heart rate variability (HRV) analysis is frequently used to describe the autonomic nervous system function. Previous studies have demonstrated the benefits of taVNS on the autonomic nervous system of healthy volunteers. For example, Clancy *et al*. demonstrated that taVNS improves cardiac autonomic regulation in healthy subjects, increases heart rate variability, and decreases sympathetic nerve outflow [15]. Antonino *et al*. demonstrated that noninvasive VNS via ABVN improved autonomic regulation in healthy young men [16]. However, these previous studies were conducted during specific time periods, such as during the morning (8:00–10:00) or afternoon (14:00–16:00), but human responses to stimuli are not always fixed. The circadian rhythm is a fundamental daily cyclical process that affects a wide range of physiological functions [17], and it is unclear whether the effects of stimulation on HRV are limited to specific time periods. Finding the optimal time of day for vagal stimulation is especially important to determine the best effect of stimulation, which has a wide range of effects on human health and work performance.

We report two studies based on these considerations to examine whether the effects of taVNS on HRV are dependent on the time of day or stimulus duration. In the first study, we measured HRV and performed taVNS at two time points, in the morning and in the evening, to compare the effects of different times on the autonomic nervous system. We also investigated whether baseline parameters could be used to predict responses to the stimuli. In the second study, we analyzed whether different durations had greater effects on HRV.

## Materials and methods

### Participants

Healthy young volunteers were recruited for this study through personal contact, and all participants voluntarily participated and provided written informed consent. All studies were approved by the ethics committee of the Hebei University of Technology and conformed to the standards outlined in the Declaration of Helsinki.

Participants were excluded if they had a history of cardiovascular disease (e.g., cardiac arrhythmia), severe inflammatory, diabetic, or epileptic disorders, and if they had recently taken any medication that might affect the autonomic nervous system. Twenty-seven participants were enrolled in Study 1 and 16 in Study 2.

### Transcutaneous auricular vagus nerve stimulation (taVNS)

taVNS was conducted using an FDA-approved percutaneous vagus nerve stimulator (taVNS device, Parasym Ltd, UK) and an associated electrode clip. Electrode clips were attached to the inner and outer surfaces of the left tragus, as prior research has mostly applied left-sided stimulation. To date, no systematic safety study has directly compared stimulation sites to examine possible adverse cardiac effects [18]. The participants applied taVNS continuously with a pulse width of 250 μs and a pulse frequency of 25 Hz.

During stimulation, the stimulus intensity was adjusted to the sensory threshold (5–35 mA) [19]. The condition of the subjects was monitored throughout the stimulation period, and the experiment was promptly terminated if adverse reactions occurred. Following stimulation, each participant completed a pain rating scale and tension rating scale to indicate whether they experienced pain or tension during the stimulation, with the scores ranging from 0 to 10 and corresponding to sentiments of “not at all” to “very much”.

### Study procedure

All the studies were conducted in a quiet laboratory environment with an indoor temperature of approximately 24°C. One week before the start of the experiment, each participant entered the laboratory to familiarize themselves with the experimental process, understand the research protocol, and receive a brief taVNS session to relieve tension during the formal experiment. To minimize external distractions, the participants sat quietly in chairs, breathed regularly and calmly, and were asked to stay awake during each experiment.

#### Study 1

Study 1 examined the effects of circadian stage stimulation on the autonomic nervous system. The morning time was set according to normal work and study hours (8:00 to 10:00), and the evening experiment time was set as two hours before one’s typical sleep (20:00 to 22:00). During the formal experiment, each participant entered the laboratory twice, in the morning and evening. Each participant was asked to complete a questionnaire on demographics such as age, weight, and height during the initial visit. Before the start of the experiment, each participant’s tragus was cleaned with an alcohol cotton swab to remove oil from their ear’s surface and to decrease skin resistance. The experimental equipment was then connected, and the participants were asked to rest for 5 min before starting the experiment. Participants wore taVNS electrodes throughout the study; however, the current was applied only during the stimulation periods, and no stimulation was received during other periods. The data were recorded at baseline, stimulation, and recovery. ECG signal acquisition was performed for 10 min (baseline period). The experimental equipment was then adjusted for 10 min of continuous electrical stimulation (stimulation period), and ECG signals were collected synchronously. Each participant received only one stimulation session in the morning, and ECG signal acquisition was performed for 10 min (recovery period) immediately following the session. This also applies to the evening session in Study 1. The order of visits was random, but the order of the recorded data was consistent.

#### Study 2

In Study 2, we explored the effect of different stimulus durations on HRV, and this study was conducted in the morning (8:00 to 10:00). The baseline and preceding procedures were identical to those used in Study 1, and these were followed by 20 min of continuous stimulation and synchronous ECG acquisition. Each participant received only one stimulation session, and ECG acquisition was performed immediately after the session for 20 min (recovery period).

### Data acquisition and analysis

The ECG signals of the participants were recorded using ECG signal acquisition equipment (PC-80B, Shanghai Lixin Instrument Co., Ltd.), and the ideal range of the sampling frequency suggested by the task force was 250–500 Hz or higher [20]; therefore, the sampling frequency in this study was set at 1024 Hz. To standardize the study, the working group also suggested that the short-term measurement analysis of HRV should be a continuous 5-min RR interval sequence (time period between successive heartbeats). Therefore, MATLAB R2018b (The MathWorks Inc., MA, USA) was used in this study to process the data. Consecutive 5-min segments were manually selected from the ECG for analysis, R-peaks were detected, and the heartbeat intervals required for HRV analysis were obtained. The ECG data were visually inspected to ensure that all R peaks of each 5-min segment were detected and that there were no abnormalities, such as ectopic beats (e.g., premature ventricular contractions), motion artifacts, missing data, and so on. HRV indicators were analyzed offline for 5 min using HRV analysis 1.0 software [21].

While the participants were free to breathe spontaneously, a flexible belt (PC-80B, Shanghai Lixin Instruments Co., Ltd.) was used to monitor and record their breathing rates. The sampling rate was set to 32 samples/s to ensure a minimum respiration rate of ≥ 10 breaths/min, because at slower breathing rates, the high frequency component of the HRV spectrum was merged with the low frequency component into a more powerful oscillation [22]. Participants were excluded if they did not achieve this level.

### HRV variables

#### Time-domain parameters

Time-domain HRV variables included: SDNN (standard deviation of all RR intervals), RMSSD (root mean square of successive RR interval differences), and pNN50 (number of continuous RR interval differences greater than 50 ms as a percentage of the total number of RR intervals). Among them, SDNN represents global autonomic regulation, which can reflect the overall variability of heart rate, and RMSSD and pNN50 reflect parasympathetic outputs [23].

#### Frequency-domain parameters

HRV was decomposed into components with different frequency ranges using a Fast Fourier Transform (FFT). Frequency-domain HRV variables included: low frequency (LF) components detected at 0.04–0.15 Hz, high frequency (HF) components detected at 0.15–0.40 Hz, and total power (TP) detected at 0.04–0.40 Hz. The low-frequency component reflects the heart rate regulation by the sympathetic and parasympathetic nerves while the high-frequency component reflects the heart rate regulation by the parasympathetic nerves, and this frequency is considered as a marker of vagal activity mediated by respiration. The ratio of low frequency to high frequency (LF/HF) components reflects the common activity level of the sympathetic and parasympathetic nerves, and a decrease in the LF/HF ratio indicates a shift in cardiac autonomic balance to decreased sympathetic activity and/or increased parasympathetic activity, thereby improving HRV [24].

#### Non-linear parameters

A Poincaré plot involves the creation of a scatter plot by comparing each RR interval with the previous interval, an important method in nonlinear research. From this, two parameters can be derived: SD1 and SD2. The standard deviation of the distance from each point to the line y = x is denoted SD1, and this act as a measure of short-term HRV and represents the parasympathetic activity. The standard deviation of the distance from each point to the line y = −x + RRm is denoted SD2, where RRm is the mean of the RR interval and represents the long and short-term variability, which is acted on by both cardiac sympathetic and parasympathetic effects [25].

#### Symbolic dynamics

The chosen RR interval sequence was spread evenly across six levels, with each level denoted by a number; in addition, a three-digit length pattern was created, transforming it into a short pattern. All patterns were classified according to the direction of change of the sequential RRs (0V, 1V, 2LV, and 2UV) and their incidence was assessed [26]. The 0V% index represents sympathetic regulation, the 1V% and 2LV% indices represent parasympathetic and sympathetic regulation, and the 2UV% index represents parasympathetic regulation [27].

### Statistical analysis

All data were statistically analyzed using IBM SPSS software (version 18). Experimental data were tested for normality using the Shapiro-Wilk test. All data were presented as group mean ± standard deviation (SD) unless otherwise stated. A two-tailed statistical test was used with a test level α = 0.05 and *p* < 0.05 indicating statistical significance.

#### Study 1

The paired-samples *t*-test (or Wilcoxon signed-rank test) was used to compare the stimulus intensity between the two groups as well as the tension and pain scores during the experiment. Repeated-measures ANOVA with post-hoc Bonferroni correction was used to analyze the time effects (baseline, stimulation, and recovery) in each group. When the data did not conform to a normal distribution, they were log-transformed or the Friedman test was performed. In addition, when the data did not meet the sphericity test criteria, a Greenhouse-Geisser correction was used.

Linear regression was used to explore the extent to which the baseline HRV indicators predicted responses to taVNS. To compare the degree of influence on the autonomic nerves between the two groups in the morning and evening, an independent sample *t*-test (or Mann-Whitney *U* test) was used to analyze the differences in the changes in HRV indexes between the baseline period and the stimulation period among the morning and evening groups. Independent sample *t*-tests (or Mann-Whitney *U* tests) were used to explore the differences in the baseline measures of various HRV indicators between responders and non-responders.

#### Study 2

Owing to the non-normal distribution of the data, the Friedman test was used. For analysis, the stimulation and recovery periods were separated into four 5-min sub-periods. Next, Friedman’s test was used to compare the baseline, stimulation, and recovery periods. Finally, a Bonferroni correction was used to detect differences in the pairwise comparisons.

## Results

### Study 1

A total of 27 healthy participants were recruited for this study. Among these, two were excluded owing to abnormal ECG signals and one was excluded because their respiratory rate was < 10 breaths per minute. Finally, 24 participants completed the experiment without experiencing any major adverse responses. Participants’ characteristics and scale scores are presented in Table 1. There was no statistically significant difference in the stimulation current intensity between the morning and evening periods (morning: 17.38 ± 4.61 mA, evening: 16.96 ± 4.62 mA, *p* = 0.364), nor, were there any statistically significant differences in the tension scores (*p* = 0.642) and pain scores (*p* = 0.576) of the participants during these periods.

**Table 1:** Characteristics and scale scores of participants.

	Morning	Evening	*p*-value
N (male/female)	27 (13/14)	—	—
Age (years)	24.08 ± 1.02	—	—
BMI (kg/m2)	22.17 ± 2.12	—	—
Intensity (mA)	17.38 ± 4.61	16.96 ± 4.62	0.364
Tension score	2.38 ± 1.06	2.5 ± 1.18	0.642
Pain score	2.33 ± 0.87	2.13 ± 0.90	0.576

Data are presented as mean ± SD, and the *p*-values from the paired *t*-test (or Wilcoxon signed-rank test) are provided.

#### Effects of morning and evening taVNS on HRV measurements

There were no statistically significant differences in the HRV baseline measures between the morning and evening groups (paired *t*-test or Wilcoxon signed-rank test, *p* > 0.05); thus, specific *p*-values were not provided. The group mean ± SD of HRV parameters for all participants are shown in Table 2.

**Table 2:** Measurements of different HRV indices between the two groups.

	Morning			Evening		
HRV index	Baseline	taVNS	*p*-value	Baseline	taVNS	*p*-value
HR	74.88 ± 7.87	72.91 ± 7.57	0.001	71.70 ± 7.71	71.01 ± 7.47	n.s.
SDNN (ms)	46.37 ± 13.25	50.95 ± 12.14	0.003	48.53 ± 13.04	52.35 ± 12.31	n.s.
RMSSD (ms)	31.72 ± 11.83	36.77 ± 11.06	< 0.001	37.57 ± 16.71	40.24 ± 16.40	0.042
pNN50 (%)	12.15 ± 13.77	18.08 ± 13.25	< 0.001	17.92 ± 17.77	20.54 ± 17.69	n.s.
Total power (ms^2^)	1870.99 ±1105.42	2364.33 ± 1142.83	0.007	1919.39 ±1305.53	2323.26 ± 1163.50	n.s.
LF power (ms^2^)	545.7 ± 396.55	624.05 ± 425.89	n.s.	591.54± 493.39	596.01 ± 425.10	n.s.
HF power (ms^2^)	314.79 ± 186.78	436.40 ± 167.55	< 0.001	426.27 ± 271.69	479.71 ± 259.71	0.028
LF/HF	2.22 ± 1.51	1.54 ± 0.82	0.043	1.72 ± 1.17	1.36 ± 0.74	n.s.
SD1	22.26 ± 8.19	26.51 ± 8.91	< 0.001	26.58 ± 11.81	28.49 ± 11.58	n.s.
SD2	60.76 ± 16.55	67.29 ± 16.64	0.004	62.51 ± 16.72	67.93 ± 15.09	n.s.
0V% (%)	24.34 ± 9.38	19.25 ± 7.28	0.007	18.81 ± 10.58	16.89 ± 6.91	n.s.
1V% (%)	49.88 ± 4.64	49.80 ± 3.42	n.s.	49.04 ± 5.81	50.43 ± 6.03	n.s.
2LV% (%)	9.95 ± 5.99	12.74 ± 6.85	0.035	12.64 ± 6.62	11.99 ± 4.61	n.s.
2UV% (%)	15.83 ± 6.93	18.20 ± 7.48	0.036	19.51 ± 10.22	20.69 ± 10.58	n.s.

Data are presented as mean ± SD, and the *p*-values from the post-hoc comparisons of the repeated-measures ANOVA (or Friedman’s test) are provided. *P*, level of significance (*p* < 0.05).

Repeated-measures ANOVA (or Friedman’s test) showed that in the morning taVNS group, measures thought to reflect vagal activity increased significantly during the stimulation session (RMSSD: *p* < 0.001; pNN50: *p* < 0.001; HF power: *p* < 0.001; SD1: *p* < 0.001; 2UV%: *p* = 0.036). Measures representing the combined action of the sympathetic and parasympathetic nerves significantly increased (SD2: *p* = 0.004), and measures representing overall HRV significantly increased (SDNN: *p* = 0.003; Total power: *p* = 0.007). In addition, the LF/HF ratio, an indicator of sympathetic and vagal balance, significantly decreased (*p* = 0.043). In the evening taVNS group, only two indicators representing vagal activity were found to be significantly increased (RMSSD: *p* = 0.042; HF power: *p* = 0.028), while other indicators did not change significantly.

#### Differences between the two groups in terms of HRV measure changes

An independent sample *t*-test (or Mann-Whitney *U* test) was performed on the magnitude of change in HRV indices during the baseline and stimulation periods to compare the difference in the degree of influence of taVNS on the HRV indexes between the two groups (Table 3). The results indicated that when taVNS was performed in the morning, the increase in the index representing the activity of the vagus nerve (RMSSD: *p* = 0.049; pNN50: *p* = 0.016; HF power: *p* = 0.003; SD1: *p* = 0.011) was significantly higher than that observed in the evening group, the difference was statistically significant, and there were no significant differences in the changes in other indicators.

**Table 3:** Changes and statistical significance of different HRV indicators between the morning and evening groups.

HRV index	Morning	Evening	*p*-value
SDNN (ms)	4.59 ± 7.72	3.82 ± 7.94	0.735
RMSSD (ms)	5.06 ± 4.62	2.67 ± 3.48	0.049
pNN50 (%)	5.93 ± 5.77	2.62 ± 5.17	0.016
HF power (ms^2^)	121.61 ± 103.93	53.45 ± 75.15	0.003
LF/HF	−0.68 ± 1.26	−0.36 ± 0.79	0.312
SD1	4.25 ± 3.42	1.92 ± 2.48	0.011
SD2	6.52 ± 12.38	5.41 ± 12.30	0.757

Data are presented as mean ± SD, and the *p*-values from the independent samples *t*-test (or Mann-Whitney *U* test) are provided. *P*, level of significance (*p* < 0.05).

#### Baseline differences between responders and non-responders

Using the definition of response proposed in the literature (with responders having a > 20% decrease in LF/HF) [15], we identified 15 responders and 9 non-responders in the morning and 9 responders and 15 non-responders in the evening. Further analysis revealed a similar pattern in the morning and evening, with significantly lower baseline measures of indicators representing vagal activity (RMSSD, HF power, SD1) in responders than in non-responders. Additionally, the baseline measures of sympathetic activity (0V%) and sympathetic-vagal balance (LF/HF) were significantly higher in responders than in non-responders (Table 4). This showed that responders had lower vagal activity and higher sympathetic activity at rest than non-responders; in addition, there was a greater number of responders when the stimulation was performed in the morning. There were no significant differences in demographic information between responders and non-responders.

**Table 4:** HRV baseline measurements and the statistical significance in responders and non-responders.

	Morning			Evening		
HRV index	Responders (n = 15)	Non-responders (n = 9)	*p*-value	Responders (n = 9)	Non-responders (n = 15)	*p*-value
HR	77.57 ± 5.66	70.40 ± 9.26	0.058	75.55 ± 4.55	69.39 ± 8.41	0.025
SDNN (ms)	42.54 ± 11.88	52.75 ± 13.57	0.066	44.21 ± 11.73	51.12 ± 13.47	0.216
RMSSD (ms)	27.40 ± 7.98	38.90 ± 14.08	0.03	29.29 ± 8.77	42.55 ± 18.55	0.021
LF power (ms^2^)	640.47± 464.08	387.73 ± 174.03	0.194	580.71 ± 328.43	598.04 ± 581.52	0.519
HF power (ms^2^)	232.40 ± 90.49	452.11 ± 228.09	0.021	230.91 ± 121.15	543.48 ± 271.34	0.004
LF/HF	2.84 ± 1.50	1.18 ± 0.81	0.006	2.61 ± 0.89	1.18 ± 0.99	0.001
SD1	19.28 ± 5.66	27.23 ± 9.61	0.025	20.74 ± 6.23	30.08 ± 13.11	0.021
0V% (%)	27.69 ± 9.28	18.75 ± 6.78	0.02	25.80 ± 12.25	14.61 ± 6.92	0.029

Data are presented as mean ± SD, and the *p*-values from the independent samples *t*-test (or Mann-Whitney *U* test) are provided. *P*, level of significance (*p* < 0.05).

#### Prediction of responses to taVNS according to baseline HRV

**Fig 1:**
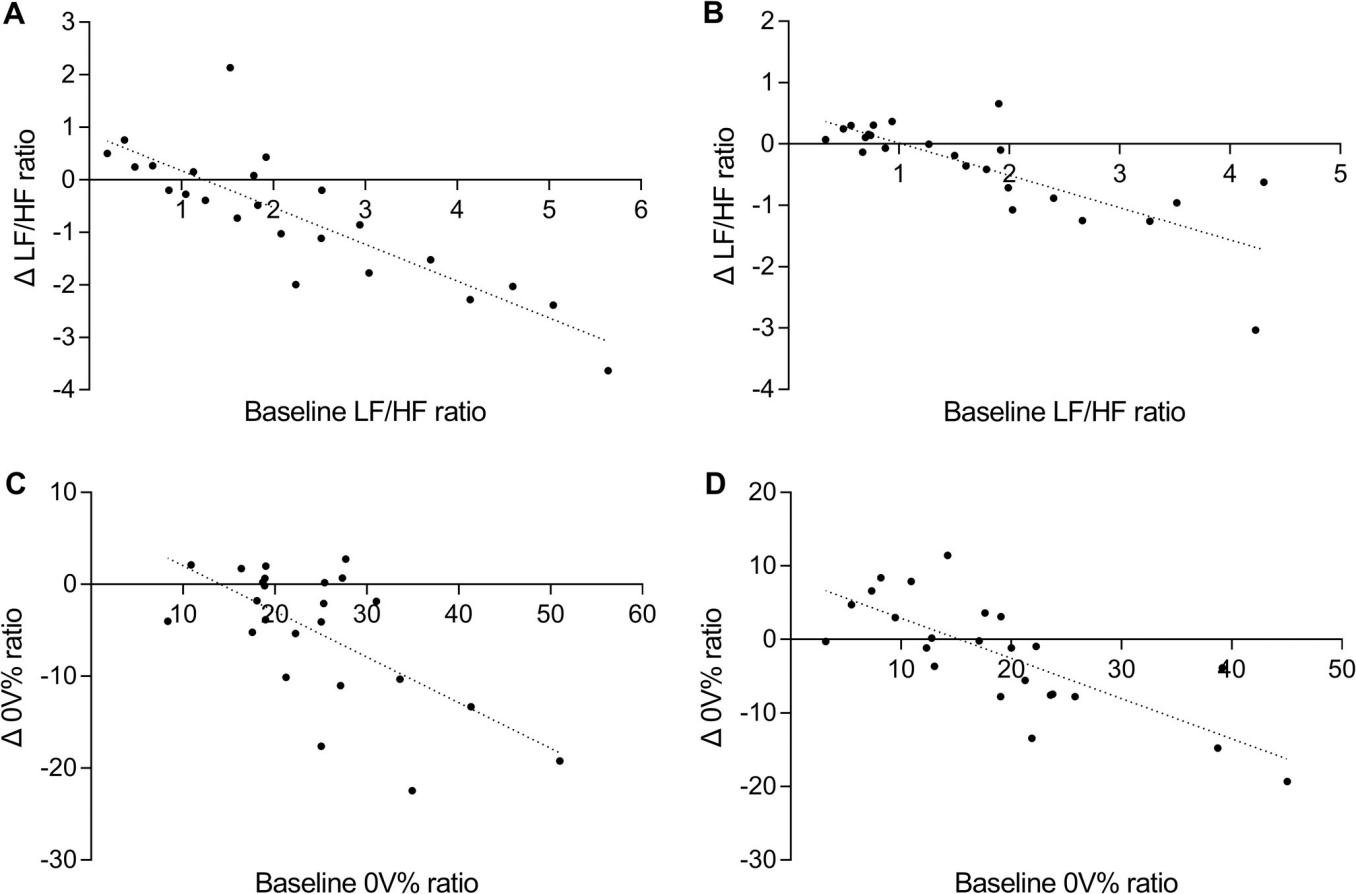
Baseline LF/HF ratio and 0V% significantly predicted changes in LF/HF and 0V% during taVNS. **A** higher baseline LF/HF ratio was associated with a greater LF/HF decline (morning: R^2^ = 0.703, *p* < 0.0001, A; evening: R^2^ = 0.6161, *p* < 0.0001, B), whereas a higher baseline 0V% was associated with a greater 0V% decline (morning: R^2^ = 0.4147, *p* = 0.0007, C; evening: R^2^ = 0.5751 *p* < 0.0001, D).

Linear regression showed the relationship between baseline HRV measurements and changes in HRV measurements during taVNS, with a higher baseline LF/HF ratio predicting a greater LF/HF decline (morning: R^2^ = 0.703, *p* < 0.0001, Fig 1A; evening: R^2^ = 0.6161, *p* < 0.0001, Fig 1B). In addition, we found a similar pattern at 0V%, where a higher baseline 0V% predicted a larger 0V% decline (morning: R^2^ = 0.4147, *p* = 0.0007, Fig 1C; evening: R^2^ = 0.5751, *p* < 0.0001, Fig 1D).

#### Carryover effect of taVNS

**Fig 2:**
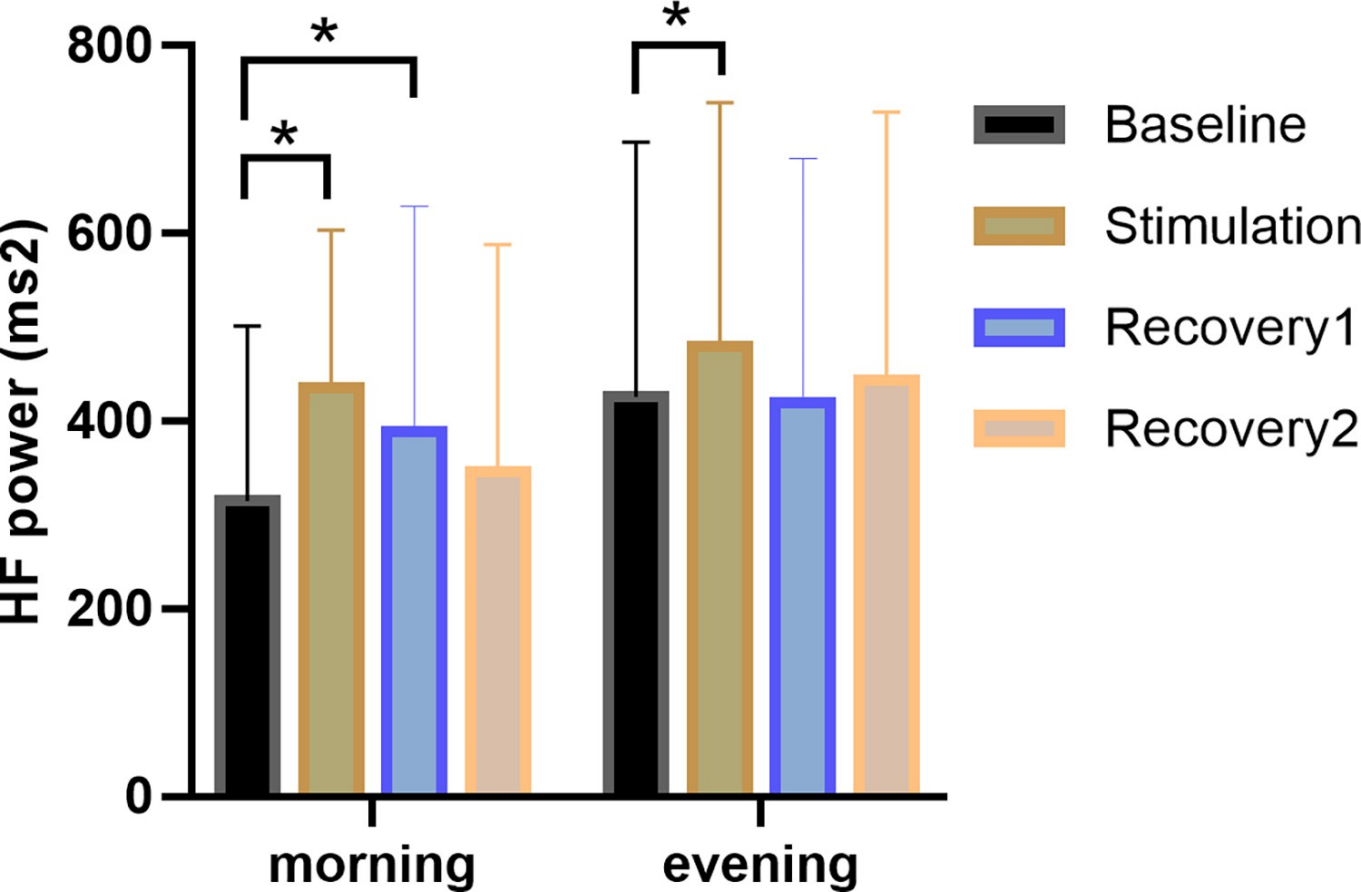
Changes in the HF component at different stimulation times. In the morning, the HF component increased significantly during stimulation and the first 5 min of recovery compared to baseline, whereas in the evening, only the stimulation period increased significantly compared to baseline. * = significantly different from baseline.

When the morning recovery phase was separated into two 5-min subphases for analysis, a carry-over effect of taVNS was observed: HF power measurements remained significantly higher than at baseline during the first 5 min of the recovery period (baseline: 314.79 ± 186.78, recovery1: 389.195 ± 240.02, *p* = 0.005). It should be noted that the same results were not observed at night (Fig 2).

### Study 2

Study 1 showed that the effect of taVNS on HRV markers was dependent on the time of day (morning *vs*. evening), and a carry-over effect was found after the stimulation ended. As a result, we conducted another study to determine whether different durations had stronger effects on HRV and whether the carry-over effect of taVNS could last longer.

Another 16 healthy volunteers (8 male, 8 female, age = 23.86 ± 1.02) participated in this study. No HR and respiratory abnormalities were observed during the experiment, and no adverse reactions occurred. Finally, all 16 participants successfully completed the experiment, and the current intensity during stimulation was 15.25 ± 3.68 mA. Table 5 shows the three HRV parameter measurements for all participants.

**Table 5:** Measurement of three HRV parameters in different periods.

Stage	RMSSD	HF power (ms^2^)	LF/HF
Baseline	31.74 ± 16.36	347.12 ± 327.74	2.16 ± 1.76
taVNS1	38.95 ± 16.86	459.9 ± 384.65	1.72 ± 0.98
taVNS2	38.48 ± 16.63	472.59 ± 345.56	2.03 ± 1.47
taVNS3	34.74 ± 14.77	434.68 ± 405.28	1.94 ± 1.26
taVNS4	36.73 ± 16.89	445.93 ± 352.50	2.36 ± 1.43
Recovery1	35.19 ± 14.68	462.73 ± 388.95	1.97 ± 1.66
Recovery2	35.36 ± 14.26	440.43 ± 345.90	2.52 ± 1.61
Recovery3	34.52 ± 14.52	407.02 ± 389.06	2.80 ± 1.53
Recovery4	33.60 ± 13.85	357.64 ± 276.99	2.46 ± 1.31

Data are presented as mean ± SD.

During the stimulation period in Study 2, the obtained 20-min continuous ECG signals were divided into four 5-min sub-phases, denoted as taVNS1, taVNS2, taVNS3, and taVNS4, respectively. This also applies to the recovery periods.

#### Effects of taVNS on HRV parameters

Friedman’s test was performed on the HRV measurements acquired during the baseline and stimulation periods. Compared to baseline, HF power (taVNS1 *vs*. baseline, *p* = 0.008; taVNS2 *vs*. baseline, *p* = 0.008) measurements increased significantly in the first two 5-min subphases of the stimulation period; however, the latter two 5-min sub-periods did not show a significant difference from the baseline period. In addition, we found the same results for the RMSSD measurements (taVNS1 *vs*. baseline, *p* < 0.001; taVNS2 *vs*. baseline, *p* = 0.003). In terms of LF/HF ratios, there were no significant differences between the stimulation and baseline periods.

We next examined the baseline and recovery periods independently, and interestingly, found a partial carry-over effect after the stimulus ended. The HF power (recovery1 *vs*. baseline, *p* = 0.012) measurements increased significantly over the first 5 min of the recovery period compared to baseline. The other parameters and sub-phases did not show the same results. Nonetheless, we found that the LF/HF ratio showed an insignificant decrease during the first 5 min of stimulation. Additionally, the RMSSD and HF power measurements increased during the first subphase of the recovery period compared with the subphase of the partial stimulation period (see Table 5). Finally, during the recovery phase, the RMSSD and HF power measurements showed a gradually decreasing trend.

## Discussion

This study demonstrated that receiving taVNS in the morning resulted in better HRV changes in healthy individuals; in addition, a significantly greater response to stimulation was observed in the morning compared to in the evening. Furthermore, the baseline LF/HF ratio and 0V% significantly predicted changes in LF/HF and 0V% during taVNS. We found no gradual increase in HRV with increased stimulation durations, suggesting that taVNS has a limited effect on HRV that is not modulated by stimulation duration. Interestingly, both studies showed a carry-over effect of stimulation, where the HF measure during the partial phase of the recovery period was significantly higher than that during the baseline period. This serves as a reminder that post-stimulus recovery time must be considered when conducting experimental studies with short time intervals to avoid carry-over effects on the results.

Many studies have reported the effects of taVNS on the HRV of healthy individuals. For example, Geng *et al*. found that taVNS can alter the HRV of healthy young adults and increase cardiac vagal activity [28]. Additionally, a study showed that taVNS increased vagal tone measurements and that daily taVNS for two weeks improved autonomic function [29]. However, several studies have presented differing views on the impact of taVNS on HRV metrics. For example, Weise *et al*. found that left tragus stimulation had no significant effect on the LF/HF ratio [30], and Clancy *et al*. showed that taVNS significantly reduced the LF/HF ratio but had no significant effect on other indicators of HRV [15]. Our findings suggest that taVNS increases cardiac vagal activity and is more effective in the morning than in the evening.

In Study 1, there was no significant difference in baseline HRV between the morning and evening periods, a finding similar to that of a previously conducted HRV circadian rhythm study [31, 32]; however, the HRV in the evening was still slightly greater than that in the morning, which may explain our conclusions. We also controlled for the effects of some confounding factors, as previous studies showed that body posture may alter autonomic control and affect sympathetic-parasympathetic balance [33]; therefore, all our studies were conducted in a sitting position. In addition, subject’s responses to stimuli are not always fixed, as they may be influenced by their current state of activity, attention, and emotion, among other factors [34, 35]. We took these into account to ensure that the stimuli were conducted in the same experimental environment and took care to alleviate participants’ anxiety about the experiment, thus increasing the comparability between the experiments.

Unlike invasive VNS, which directly stimulates the efferent cardiac branches of the vagus nerve, the auricular branch of the vagus nerve consists only of afferent fibers, which have no afferent nerves directly connected to the heart and only indirectly modulate these through the brainstem nuclei; therefore, the effects of taVNS on HRV are all indirect. Specifically, as proposed by Murray and colleagues [9], taVNS may increase the level of input to the nucleus tractus solitarius (NTS), thereby increasing the activity of NTS neurons projecting to the two vagal efferent nuclei, and further activating the dorsal motor nucleus of the vagus and the nucleus ambiguous to increase cardiac parasympathetic activity.

In Study 1, the same pattern was found in both the morning and evening, with higher LF/HF ratios and 0V% predicting larger LF/HF ratios and 0V% declines. These results indicated that HRV can be significantly improved by taVNS when the cardiac autonomic balance is biased toward sympathetic innervation. This conclusion is similar to that of previous studies, and predictions from baseline HRV measurements could be used to screen for optimal taVNS subjects [36]. In addition, the pre-onset of severe arrhythmias is characterized by an increase in the 0V pattern, and the sign dynamics of the RR series can predict life-threatening arrhythmias [37]. Therefore, combining the above two indicators for prediction is critical for the adoption of effective treatment during the early stages of the disease [38, 39].

Similar to the study by De Couck *et al*. [40], our study found that HRV parameters did not increase with stimulation duration. However, RMSSD and HF power measurements were significantly higher in the first 10-min of stimulation compared to during the baseline period, which may aid in determining the optimal stimulation duration. Interestingly, we discovered that regardless of whether the stimulation was 10 or 20 min, there was a certain stimulation carry-over effect in the recovery period after the stimulation ended, which showed a downward trend with time. Similar conclusions have been reached in previous studies. For example, during the recovery period following taVNS cessation, both the LF/HF ratio and muscle sympathetic nerve activity remained lower than at baseline [15]. Furthermore, an fMRI investigation of electrical stimulation of the outer ear showed that the activation of the nucleus tractus solitarius gradually decreased over time after the end of stimulation, a process that lasted 11 min [41]. Although the statistical analysis showed that only HF power measurements were significantly higher than baseline during the first 5 min of the recovery period, the RMSSD and HF power remained higher than baseline during the other sub-phases of the recovery period. Furthermore, the LF/HF ratio was lower during the first 5 min of the recovery phase than during the baseline period. We could only demonstrate that the stimulation had a carry-over impact and that the effect was best 5 min after the stimulation ended. Of course, this may be due to the small sample size of the study; therefore, future studies should consider extending the sample size and further increasing the stimulation time to examine the carry-over effect of taVNS.

This study has several limitations. First, sham stimulation was not included in this study as the aim was to examine whether the effect of taVNS on HRV was dependent on the time of day or duration of stimulation. Furthermore, as the participants were not taking any medications that could affect their autonomic nervous systems, it is unclear whether taVNS could also affect HRV when used in combination with medications. Second, all of our study subjects were healthy young college students who had regular work and rest schedules; thus, inferences pertaining to results in other populations (such as in middle-aged and elderly people, patients with diseases, people working in different occupations, or those working night shifts) were not possible, and these populations may have varying autonomic neuromodulation abilities. Future studies should consider these populations further. Third, the indicators measured in this study were HRV parameters. Although some conclusions have been drawn, future research should attempt to combine HRV parameters with other indicators to obtain more precise results [42]. Fourth, there is evidence that stimulation frequency and intensity can affect the extent of taVNS activation [43, 44]; therefore, it is unclear whether the effects of stimulation timing and duration vary with other stimulation parameters. It is worth noting that the stimulation intensity used in this study was much higher than that used in the taVNS study of cymba concha, which may be the reason for the conflicting results of taVNS on HRV in the literature. Furthermore, fluctuations in breathing rates may affect HRV parameters, even when participants’ breathing is monitored.

## Conclusions

taVNS was more effective in the morning, the effect of stimulation on HRV was not modulated by the duration of stimulation, and the best results appeared to be achieved within 10 min. Although there was a carry-over effect following stimulation, both of our studies found that only the measurements acquired within 5 min were significantly higher than the baseline values. In the future, the sample size should be expanded to further explore the carry-over effect after the end of stimulation, find the applicable population of taVNS, and address the limitations of this study.
